# Associations between underlying diseases with COVID-19 and its symptoms among adults: a cross-sectional study

**DOI:** 10.3389/fpubh.2023.1210800

**Published:** 2023-06-13

**Authors:** Binghan Wang, Shuyan Yuan, Shuke Ruan, Xiuyuan Ning, Hanrui Li, Yuanhao Liu, Xiuyang Li

**Affiliations:** ^1^Department of Big Data in Health Science, and Center for Clinical Big Data and Statistics, the Second Affiliated Hospital, College of Medicine, Zhejiang University, Hangzhou, China; ^2^School of Public Health, Zhejiang University School of Medicine, Hangzhou, China

**Keywords:** underlying diseases, COVID-19, severe symptoms, loss of sensory, cross-sectional study

## Abstract

**Background:**

Specific underlying diseases were reported to be associated with severe COVID-19 outcomes, but little is known about their combined associations. The study was aimed to assess the relations of number of and specific underlying diseases to COVID-19, severe symptoms, loss of smell, and loss of taste.

**Methods:**

A total of 28,204 adult participants in the National Health Interview Survey 2021 were included. Underlying diseases (including cardiovascular diseases, cancer, endocrine diseases, respiratory diseases, neuropsychiatric diseases, liver and kidney diseases, fatigue syndrome, and sensory impairments), the history of COVID-19, and its symptoms were self-reported by structured questionnaires. Multivariable logistic regression models were used to assess the combined relation of total number of underlying diseases to COVID-19 and its symptoms, while mutually adjusted logistic models were used to examine their independent associations.

**Results:**

Among the 28,204 participants (mean ± standard deviation: 48.2 ± 18.5 years), each additional underlying disease was related to 33, 20, 37, and 39% higher odds of COVID-19 (odds ratio [OR]: 1.33, 95% confidence interval [CI]: 1.29–1.37), severe symptoms (OR: 1.20, 95% CI: 1.12–1.29), loss of smell (OR: 1.37, 95% CI: 1.29–1.46), and loss of taste (OR: 1.39, 95% CI: 1.31–1.49). In addition, independent associations of sensory impairments with COVID-19 (OR: 3.73, 95% CI: 3.44–4.05), severe symptoms (OR: 1.37, 95% CI: 1.13–1.67), loss of smell (OR: 8.17, 95% CI: 6.86–9.76), and loss of taste (OR: 6.13, 95% CI: 5.19–7.25), cardiovascular diseases with COVID-19 (OR: 1.13, 95% CI: 1.03–1.24), neuropsychiatric diseases with severe symptoms (OR: 1.41, 95% CI: 1.15–1.74), and endocrine diseases with loss of taste (OR: 1.28, 95% CI: 1.05–1.56) were observed.

**Conclusion:**

A larger number of underlying diseases were related to higher odds of COVID-19, severe symptoms, loss of smell, and loss of taste in a dose–response manner. Specific underlying diseases might be individually associated with COVID-19 and its symptoms.

## Introduction

Coronavirus disease 2019 (COVID-19) is a global pandemic caused by the severe acute respiratory syndrome coronavirus (1, 2), which can lead to multiple clinical outcomes, from asymptomatic infection to severe infection and even death (3–5). COVID-19 patients with underlying diseases (e.g., cardiovascular disease, diabetes, liver disease, and lung disease) were identified as particularly vulnerable populations, may have worse COVID-19 outcomes and a greater mortality (6). A research estimates that 1.7 billion people (22% of the global population) have at least one underlying disease, who might have higher odds of developing serious symptoms after contracting SARS-CoV-2 (7). The existence of underlying diseases implies functional decline of the corresponding systems, indicating that individuals are in poor health and an increased risk of COVID-19 (8). Lacking knowledge about novel variants and insufficient vaccination (especially in high-risk populations), COVID-19 remains dangerous and continues to cause a significant personal and social healthcare burden (9, 10).

Previous population-based studies had reported the associations of individual underlying diseases with COVID-19 and its symptoms (11–13). For example, an umbrella review reported diabetes, heart failure, chronic obstructive pulmonary diseases, and dementia were related to higher risk of fatal COVID-19 (hazard ratio ranged from 1.2 to 7.7) (13). Using comorbidity indexes, some studies examined overall impact of comorbidities in hospitalized patients, but the approach eliminated specific system information and general population evidence remain relatively scarce (14, 15). Additionally, the types of underlying diseases covered by the currently available studies were not comprehensive (e.g., mental disease and sensory impairment were not included). Considering that the functions of human systems interact with each other (16), it is necessary to explore these relations from a holistic perspective by linking the diseases of each system.

In order to advance this field of study and offer fresh perspectives on the pathogenesis and treatment of COVID-19 disease in the context of underlying diseases, data from the National Health Interview Survey (NHIS) 2021 was used to examine the independent and combined associations of underlying diseases with COVID-19 and its symptoms.

## Methods

### Study population

The NHIS is an annual survey conducted by the US Centers for Disease Control and Prevention (CDC) with a national representative of the noninstitutionalized US population. It selects a sample of dwelling units using geographically clustered sampling techniques. Each randomly selected family has an adult and a child interviewed over the telephone or in person. More detailed information can be found elsewhere.^1^ In 2021, NHIS collected information on demographics, health insurance, medical access and use, health status, tobacco use, function and disability, and the history of COVID-19 and its symptoms for adults. The NHIS was approved by the research ethics review board of the National Center for Health Statistics of the US, Centers for Disease Control and Prevention (CDC). Oral or written informed consent was provided by all participants.

Among 29,482 NHIS adult participants (aged ≥18) in 2021, 191 participants without information on COVID-19 and 1,087 participants with missing data on underlying diseases were excluded. The analysis finally included 28,204 participants in the NHIS.

### Assessments of underlying diseases

Self-reported underlying diseases were collected through a structured questionnaire, including cardiovascular diseases (hypertension, coronary heart disease, angina, heart attack, and stroke), cancer, endocrine diseases (diabetes and high cholesterol), respiratory diseases (asthma, chronic obstructive pulmonary diseases [COPD], emphysema, and chronic bronchitis), neuropsychiatric diseases (dementia, epilepsy, depression, and anxiety), liver and kidney diseases (weak or failing kidneys, hepatitis, cirrhosis, and liver conditions), fatigue syndrome, and sensory impairments (visual, hearing, olfactory, and taste impairments). The total number of underlying diseases ranged from 0 to 8. Participants were asked whether they had ever been told by a doctor or other health professional that they had a certain disease. Individuals who answered yes were considered to have the corresponding diseases. Additionally, self-reported difficulties in seeing, hearing, smelling, and tasting were used to define sensory impairments (17–19).

### Assessments of history of COVID-19, severe symptoms, loss of smell, and loss of taste

In NHIS 2021, self-reported information was collected on the history of COVID-19, severity of post-infection symptoms, and loss of smell and taste. Participants who were received a diagnosis of COVID-19 from doctors or other health professionals or had tested positive for COVID-19 were deemed to have a history of COVID-19, and they were further asked about the symptoms of COVID-19. Participants reported ‘severe symptoms’ defined as severe symptoms.

### Other covariates

Covariate data were collected by structured questionnaires in the NHIS, including age (continuous variable, years), gender (male/female), race (Hispanic/Non-Hispanic White/Non-Hispanic Black/other races), education (below college/college and above), family income-poverty ratio (<1.5/1.5–3.5/>3.5), household region (Northeast/Midwest/South/West), health insurance hierarchy (Private/Medicaid and other public/other coverage/uninsured), marital status (married or partnered/other statuses), ever smoking (yes/no), body mass index (BMI, <25/25- < 30/> = 30 kg/m^2^), history of pneumonia shot (yes/no) and flu vaccine in the past 12 months (yes/no), and interview month (from Jan. to Dec.).

### Statistical analyses

The mean ± standard deviation (SD) for normally distributed continuous data and the relative number for categorical variables were presented after standardization according to weights given by NHIS. Using the “mice” package (20), multiple imputations were conducted to handle the presence of missing values of the covariates.

To explore the association of comorbidity of underlying diseases with COVID-19 and its symptoms, the Student’s t test and Chi-square test were used to univariable analysis of COVID-19 and its symptoms. Logistic regression models were used to evaluate the odds ratio (OR) and 95% confidence intervals (CIs). Multivariable models were adjusted for socioeconomic factors (including age, gender, race, education, family income-poverty ratio, marital status, household region, and interview month), lifestyle factors (including smoking status and BMI), and health related behaviors (including health insurance hierarchy and history of pneumonia shot and flu vaccine). In analyses of the number of underlying diseases, participants without any underlying diseases were defined as the reference group. Moreover, the *P*-trend was calculated by allowing the number of underlying diseases to be a continuous variable. To further assess the independent associations of specific underlying diseases with COVID-19 and its symptoms, each disease was individually included in the multivariable adjusted models, and then the eight underlying diseases were included in the mutually adjusted models simultaneously to assess the independence of each component. Additionally, an exploratory analysis was conducted to evaluating the associations of dyads of underlying diseases with COVID-19, adjusted for covariates as in the main analyses and the presence of other underlying diseases. The reference group was also made up of participants without any underlying diseases.

Furthermore, subgroup analyses were conducted by age (<65/> = 65 years), gender (male/female), race (Hispanic/Non-Hispanic White/Non-Hispanic Black/other races), education (below college/college and above), family income-poverty ratio (<1.5/1.5–3.5/>3.5), and marital status (married or partnered/other statuses).

All analyses were conducted by R (version 4.2.2, http://www.R-project.org). Two-sided *P*-values and 95% confidence intervals were reported throughout, and *P*-value less than or equal to 0.05 represented statistical significance.

## Results

### Participants characteristics

Among 28,204 NHIS participants, 51.7% were female, 63.2% were non-Hispanic White, 37.3% had education level lower than college degree, 49.9% had family income-poverty ratio higher than 3.5, 60.2% were married or partnered, 34.9% had ever smoked, 32.7% were obese, 37.9% lived in the South, 90.1% had health insurance, 48.9% had received an influenza vaccination in the past 12 months, 23.9% had history of pneumonia vaccination, and the weighted mean (SD) age was 48.2 (18.5) years (Table 1). Moreover, among 12.8% of participants with history of COVID-19, 18.5% had severe symptoms, 59.1% lost the sense of smell, and 57.0% lost the sense of taste.

**Table 1:** Characteristics of the study participants in the National Health Interview Survey.

Characteristics		Characteristics	
*Age, years, mean* ± *SD*	48.2 ± 18.5	*Household region, %*	
*Female, %*	51.7	Northeast	17.3
*Race, %*		Midwest	20.9
Hispanic	16.7	South	37.9
Non-Hispanic White	63.2	West	23.9
Non-Hispanic Black	11.6	*Health insurance hierarchy, %*	
Other races	8.5	Private	62.3
*With college degree, %*	62.7	Medicaid and other public	22.8
*Family income-poverty ratio, %*		Other coverage	5.0
<1.5	18.7	Uninsured	9.9
1.5–3.5	31.3	*Flu vaccine in the past 12 months, %*	48.9
>3.5	49.9	*Ever had pneumonia shot, %*	23.9
*Married/Partnered, %*	60.2	*Sensory impairments, %*	34.4
*Ever smoking, %*	34.6	*Cardiovascular diseases, %*	33.9
*Body mass index, %*		*Endocrine diseases, %*	30.7
<25 kg/m^2^	33.2	*Neuropsychiatric diseases, %*	24.5
25 – <30 kg/m^2^	34.1	*Respiratory diseases, %*	16.6
> = 30 kg/m^2^	32.7	*Cancer, %*	9.9
*Interview month, %*		*Liver and kidney diseases, %*	4.9
January	8.2	*Fatigue Syndrome, %*	1.3
February	8.0	*Number of diseases, %*	
March	8.7	0	28.1
April	8.4	1	27.2
May	8.3	2	21.0
June	8.3	3	13.0
July	8.7	4 or more	10.8
August	8.6	*History of COVID-19, %*	12.8
September	7.6	*Severe symptoms, n (%)*	18.5
October	8.6	*Loss of smell, n (%)*	59.1
November	8.2	*Loss of taste, n (%)*	57.0
December	8.2		

Mean and percentages presented are weighted according to the weight given by National Health Interview Survey.

**Figure 1:**
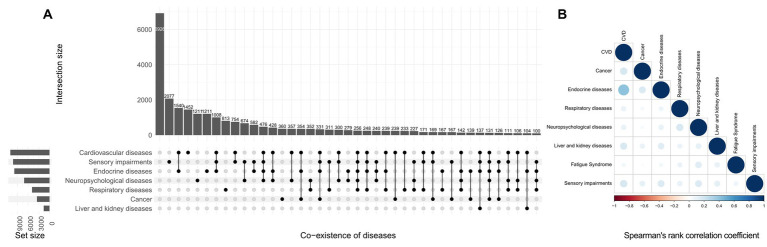
Co-existing pattern **(A)** and correlation **(B)** of diseases in the National Health Interview Survey. The size of the circle on the right represents the *P*-value, while the depth of the circle’s color represents the size of the Spearman’s rank correlation coefficient. The absolute value of the correlation coefficient is closest to 1 when the color is darker, and a wider circle denotes a smaller *P*-value.

In NHIS participants, 28.1% had no underlying disease, and 27.2, 21.0, 13.0, and 10.8% had one, two, three, and more than three diseases, respectively. The prevalence of cardiovascular diseases, cancer, endocrine diseases, respiratory diseases, neuropsychiatric diseases, liver and kidney diseases, fatigue syndrome, and sensory impairments were 33.9% (95% CI: 33.4–34.5%), 9.9% (95% CI: 9.6–10.3%), 30.7% (95% CI:30.1–31.3%), 16.6% (95% CI:16.2–17.1%), 24.5% (95% CI: 24.0–25.1%), 4.9% (95% CI: 4.7–5.2%), 1.3% (95%CI: 1.2–1.5%), 34.4% (95% CI: 33.9–35.0%). The pattern of co-existence of eight underlying diseases with their minor to moderate correlations are presented in the Figure 1.

### Univariable analyses for basic characteristics and diseases with COVID-19 and its symptoms

Table 2 presents the univariable analyses for basic characteristics and diseases with COVID-19 and its symptoms. Participants with history of COVID-19 were more likely to have respiratory diseases (*χ*^2^ = 4.16, *P* = 0.041), neuropsychiatric diseases (*χ*^2^ = 27.28, *P* < 0.001), fatigue syndrome (*χ*^2^ = 6.51, *P* = 0.011), and sensory impairments (*χ*^2^ = 876.57, *P* < 0.001). In term of severe symptoms, individuals trended to have all underlying diseases (*P* < 0.05) except for fatigue syndrome. Compared to participants without loss of smell, the prevalence of cardiovascular diseases (*χ*^2^ = 5.23, *P =* 0.022), cancer (*χ*^2^ = 15.36, *P* < 0.001), and sensory impairments (*χ*^2^ = 562.14, *P* < 0.001) were higher in those who lost sense of smell. The prevalence of cancer (*χ*^2^ = 6.78, *P* = 0.009), neuropsychiatric diseases (*χ*^2^ = 5.98, *P* = 0.014), and sensory impairments (*χ*^2^ = 475.75, *P* < 0.001) were also higher in participants who lost their sense of taste than in those without taste loss. The results of univariable analyses of other covariates can be found in Table 2.

**Table 2:** Univariable analyses of COVID-19 history and its symptoms in the National Health Interview Survey.

Characteristics	COVID-19 history		t/*χ*^2^	*p*	Severe symptoms		t/*χ*^2^	*p*	Loss of smell		t/*χ*^2^	*p*	Loss of taste		t/*χ*^2^	*p*
	No	Yes			No	Yes			No	Yes			No	Yes		
*Age, years, mean* ± *SD*	53.2 ± 18.4	47.8 ± 17.3	16.47	<0.001	46.6 ± 17.3	52.4 ± 16.5	−7.69	<0.001	50.5 ± 17.9	45.6 ± 16.5	7.89	<0.001	49.8 ± 18.0	46.0 ± 16.5	6.16	<0.001
*Female, %*	13,629 (54.5)	1777 (55.7)	1.60	0.206	1,421 (55.4)	355 (57.1)	0.50	0.478	692 (51.7)	1,072 (58.9)	15.70	<0.001	723 (51.6)	1,047 (59.0)	16.81	<0.001
*Race, %*			162.01	<0.001			3.87	0.276			21.82	<0.001			6.88	0.076
Hispanic	3,201 (12.8)	658 (20.6)			523 (20.4)	134 (21.5)			226 (16.9)	430 (23.6)			262 (18.7)	393 (22.1)		
Non-Hispanic White	16,900 (67.6)	2015 (63.1)			1,635 (63.7)	378 (60.8)			877 (65.5)	1,112 (61.1)			898 (64.1)	1,106 (62.3)		
Non-Hispanic Black	2,656 (10.6)	321 (10.1)			246 (9.6)	74 (11.9)			143 (10.7)	175 (9.6)			144 (10.3)	176 (9.9)		
Other races	2,256 (9.0)	197 (6.2)			161 (6.3)	36 (5.8)			92 (6.9)	104 (5.7)			96 (6.9)	100 (5.6)		
*With college degree, %*	16,881 (67.5)	2039 (63.9)	16.36	<0.001	1,687 (65.8)	350 (56.3)	19.18	<0.001	855 (63.9)	1,165 (64.0)	<0.01	0.996	897 (64.1)	1,135 (63.9)	<0.01	0.970
*Family income-poverty ratio, %*			11.26	0.004			38.84	<0.001			16.83	<0.001			6.54	0.038
<1.5	4,606 (18.4)	651 (20.4)			476 (18.6)	173 (27.8)			229 (17.1)	413 (22.7)			262 (18.7)	384 (21.6)		
1.5–3.5	7,747 (31.0)	1,016 (31.8)			801 (31.2)	214 (34.4)			424 (31.7)	578 (31.7)			435 (31.1)	574 (32.3)		
>3.5	12,660 (50.6)	1,524 (47.8)			1,288 (50.2)	235 (37.8)			685 (51.2)	830 (45.6)			703 (50.2)	817 (46.0)		
*Married/Partnered, %*	13,270 (53.1)	1801 (56.4)	12.92	<0.001	1,461 (57.0)	338 (54.3)	1.29	0.256	722 (54.0)	1,060 (58.2)	5.49	0.019	750 (53.6)	1,043 (58.8)	8.36	0.004
*Ever smoking, %*	9,597 (38.4)	1,116 (35.0)	13.70	<0.001	860 (33.5)	255 (41.0)	11.95	0.001	463 (34.6)	634 (34.8)	0.01	0.932	484 (34.6)	622 (35.0)	0.06	0.811
*Body mass index, %*			56.87	<0.001			45.29	<0.001			7.94	0.019			11.89	0.003
<25 kg/m^2^	8,546 (34.2)	898 (28.1)			783 (30.5)	114 (18.3)			390 (29.1)	498 (27.3)			408 (29.1)	485 (27.3)		
25 – <30 kg/m^2^	8,596 (34.4)	1,116 (35.0)			896 (34.9)	220 (35.4)			493 (36.8)	615 (33.8)			522 (37.3)	590 (33.2)		
> = 30 kg/m^2^	7,871 (31.5)	1,177 (36.9)			886 (34.5)	288 (46.3)			455 (34.0)	708 (38.9)			470 (33.6)	700 (39.4)		
*Interview month, %*			119.37	<0.001			21.75	0.026			12.70	0.313			8.22	0.694
January	2,185 (8.7)	185 (5.8)			162 (6.3)	23 (3.7)			80 (6.0)	104 (5.7)			79 (5.6)	105 (5.9)		
February	2,103 (8.4)	215 (6.7)			175 (6.8)	40 (6.4)			94 (7.0)	119 (6.5)			99 (7.1)	116 (6.5)		
March	2,273 (9.1)	236 (7.4)			205 (8.0)	31 (5.0)			108 (8.1)	128 (7.0)			119 (8.5)	117 (6.6)		
April	1980 (7.9)	218 (6.8)			176 (6.9)	42 (6.8)			92 (6.9)	125 (6.9)			87 (6.2)	130 (7.3)		
May	1941 (7.8)	219 (6.9)			181 (7.1)	38 (6.1)			105 (7.8)	112 (6.2)			97 (6.9)	121 (6.8)		
June	1920 (7.7)	235 (7.4)			177 (6.9)	56 (9.0)			105 (7.8)	127 (7.0)			108 (7.7)	122 (6.9)		
July	2,250 (9.0)	291 (9.1)			239 (9.3)	51 (8.2)			115 (8.6)	173 (9.5)			124 (8.9)	166 (9.4)		
August	2,167 (8.7)	293 (9.2)			232 (9.0)	61 (9.8)			119 (8.9)	168 (9.2)			120 (8.6)	171 (9.6)		
September	1989 (8.0)	273 (8.6)			218 (8.5)	55 (8.8)			105 (7.8)	166 (9.1)			117 (8.4)	156 (8.8)		
October	2,174 (8.7)	350 (11.0)			279 (10.9)	71 (11.4)			153 (11.4)	193 (10.6)			158 (11.3)	190 (10.7)		
November	2070 (8.3)	330 (10.3)			255 (9.9)	74 (11.9)			119 (8.9)	206 (11.3)			141 (10.1)	187 (10.5)		
December	1961 (7.8)	346 (10.8)			266 (10.4)	80 (12.9)			143 (10.7)	200 (11.0)			151 (10.8)	194 (10.9)		
*Household region, %*			22.64	<0.001			2.38	0.497			3.46	0.325			9.01	0.029
Northeast	4,070 (16.3)	454 (14.2)			365 (14.2)	89 (14.3)			205 (15.3)	247 (13.6)			217 (15.5)	236 (13.3)		
Midwest	5,363 (21.4)	710 (22.3)			581 (22.7)	128 (20.6)			304 (22.7)	398 (21.9)			311 (22.2)	395 (22.3)		
South	9,018 (36.1)	1,260 (39.5)			998 (38.9)	261 (42.0)			507 (37.9)	741 (40.7)			516 (36.9)	737 (41.5)		
West	6,562 (26.2)	767 (24.0)			621 (24.2)	144 (23.2)			322 (24.1)	435 (23.9)			356 (25.4)	407 (22.9)		
*Health insurance hierarchy, %*			59.29	<0.001			35.61	<0.001			14.66	0.002			2.19	0.533
Private	15,050 (60.2)	2093 (65.6)			1744 (68.0)	349 (56.1)			900 (67.3)	1,180 (64.8)			924 (66.0)	1,162 (65.5)		
Medicaid and other public	6,520 (26.1)	669 (21.0)			487 (19.0)	179 (28.8)			277 (20.7)	380 (20.9)			299 (21.4)	363 (20.5)		
Other coverage	1,538 (6.1)	147 (4.6)			112 (4.4)	34 (5.5)			70 (5.2)	72 (4.0)			64 (4.6)	81 (4.6)		
Uninsured	1905 (7.6)	282 (8.8)			222 (8.7)	60 (9.6)			91 (6.8)	189 (10.4)			113 (8.1)	169 (9.5)		
*Flu vaccine in the past 12 months, %*	13,483 (53.9)	1,483 (46.5)	62.42	<0.001	1,200 (46.8)	281 (45.2)	0.46	0.499	661 (49.4)	807 (44.3)	7.82	0.005	676 (48.3)	800 (45.1)	3.12	0.077
*Ever had pneumonia shot, %*	7,562 (30.2)	742 (23.3)	66.02	<0.001	557 (21.7)	184 (29.6)	16.92	<0.001	363 (27.1)	363 (19.9)	22.16	<0.001	364 (26.0)	371 (20.9)	11.15	0.001
*Cardiovascular diseases, %*	9,801 (39.2)	1,187 (37.2)	4.61	0.032	885 (34.5)	299 (48.1)	38.89	<0.001	525 (39.2)	641 (35.2)	5.23	0.022	531 (37.9)	643 (36.2)	0.90	0.342
*Cancer, %*	3,211 (12.8)	325 (10.2)	17.92	<0.001	241 (9.4)	83 (13.3)	8.12	0.004	167 (12.5)	149 (8.2)	15.36	<0.001	164 (11.7)	157 (8.8)	6.78	0.009
*Endocrine diseases, %*	8,845 (35.4)	1,026 (32.2)	12.67	<0.001	756 (29.5)	267 (42.9)	40.95	<0.001	445 (33.3)	563 (30.9)	1.84	0.175	441 (31.5)	578 (32.6)	0.36	0.549
*Respiratory diseases, %*	4,304 (17.2)	596 (18.7)	4.16	0.041	447 (17.4)	147 (23.6)	12.31	<0.001	256 (19.1)	331 (18.2)	0.41	0.524	266 (19.0)	327 (18.4)	0.14	0.712
*Neuropsychiatric diseases, %*	6,195 (24.8)	927 (29.1)	27.28	<0.001	693 (27.0)	233 (37.5)	25.98	<0.001	373 (27.9)	543 (29.8)	1.32	0.251	375 (26.8)	547 (30.8)	5.98	0.014
*Liver and kidney diseases, %*	1,439 (5.8)	190 (6.0)	0.18	0.676	129 (5.0)	61 (9.8)	19.54	<0.001	80 (6.0)	108 (5.9)	0.00	1.000	77 (5.5)	112 (6.3)	0.78	0.378
*Fatigue Syndrome, %*	346 (1.4)	63 (2.0)	6.51	0.011	48 (1.9)	15 (2.4)	0.50	0.479	24 (1.8)	38 (2.1)	0.21	0.648	23 (1.6)	40 (2.3)	1.20	0.273
*Sensory impairments, %*	8,307 (33.2)	1914 (60.0)	876.57	<0.001	1,485 (57.9)	426 (68.5)	22.96	<0.001	480 (35.9)	1,416 (77.8)	562.14	<0.001	542 (38.7)	1,366 (77.0)	475.75	<0.001
*Number of diseases, %*			161.67	<0.001			99.62	<0.001			90.31	<0.001			110.06	<0.001
0	6,433 (25.7)	493 (15.4)			439 (17.1)	53 (8.5)			302 (22.6)	187 (10.3)			320 (22.9)	172 (9.7)		
1	6,313 (25.2)	906 (28.4)			782 (30.5)	124 (19.9)			361 (27.0)	541 (29.7)			390 (27.9)	512 (28.8)		
2	5,372 (21.5)	791 (24.8)			626 (24.4)	165 (26.5)			305 (22.8)	481 (26.4)			318 (22.7)	470 (26.5)		
3	3,693 (14.8)	527 (16.5)			397 (15.5)	129 (20.7)			194 (14.5)	325 (17.8)			194 (13.9)	328 (18.5)		
4 or more	3,202 (12.8)	474 (14.9)			321 (12.5)	151 (24.3)			176 (13.2)	287 (15.8)			178 (12.7)	293 (16.5)		

### Multivariable analyses for number of and specific diseases with COVID-19 and its symptoms

In NHIS, participants with more underlying diseases were more likely to have history of COVID-19 and severe symptoms, and to lose their sense of smell and taste (Table 3). Each additional underlying disease was associated with higher odds of COVID-19 (OR: 1.33, 95% CI: 1.29–1.37, *P*-trend <0.001), severe symptoms (OR: 1.20, 95% CI: 1.12–1.29, *P*-trend <0.001), loss of smell (OR: 1.37, 95% CI: 1.29–1.46, *P*-trend <0.001), and loss of taste (OR: 1.39, 95% CI: 1.31–1.49, *P*-trend <0.001). Compared to those without any underlying disease, participants who had one, two, three, and more than three diseases were related to 121% (OR: 2.21, 95% CI: 1.97–2.49), 181% (OR: 2.81, 95% CI: 2.48–3.19), 239% (OR: 3.39, 95% CI: 2.94–3.91), 305% (OR: 4.05, 95% CI: 3.47–4.73) higher odds of COVID-19, respectively. Additionally, the number of underlying diseases showed similar dose–response associations with severe symptoms, loss of smell, and loss of taste.

**Table 3:** Associations of numbers of diseases with COVID-19 and its symptoms in the National Health Interview Survey.

Number of diseases	*β*	Standard error	Wald *χ*2	OR [95% CI]	*P*-value
*COVID-19 history*					
0 (Reference)					
1	**0.793**	**0.060**	**13.203**	**2.21 [1.97–2.49]**	**<0.001**
2	**1.035**	**0.064**	**16.196**	**2.81 [2.48–3.19]**	**<0.001**
3	**1.221**	**0.073**	**16.716**	**3.39 [2.94–3.91]**	**<0.001**
4 or more	**1.400**	**0.079**	**17.810**	**4.05 [3.47–4.73]**	**<0.001**
Every one more disease	**0.282**	**0.015**	**18.726**	**1.33 [1.29–1.37]**	**<0.001**
*Severe symptoms*					
0 (Reference)					
1	0.245	0.178	1.375	1.28 [0.91–1.82]	0.169
2	**0.631**	**0.177**	**3.574**	**1.88 [1.34–2.68]**	**<0.001**
3	**0.716**	**0.189**	**3.782**	**2.05 [1.42–2.98]**	**<0.001**
4 or more	**0.956**	**0.197**	**4.862**	**2.60 [1.78–3.84]**	**<0.001**
Every one more disease	**0.185**	**0.035**	**5.221**	**1.20 [1.12–1.29]**	**<0.001**
*Loss of smell*					
0 (Reference)					
1	**1.047**	**0.120**	**8.698**	**2.85 [2.25–3.61]**	**<0.001**
2	**1.280**	**0.128**	**9.997**	**3.60 [2.80–4.63]**	**<0.001**
3	**1.524**	**0.147**	**10.391**	**4.59 [3.45–6.13]**	**<0.001**
4 or more	**1.665**	**0.158**	**10.533**	**5.29 [3.88–7.22]**	**<0.001**
Every one more disease	**0.314**	**0.032**	**9.823**	**1.37 [1.29–1.46]**	**<0.001**
*Loss of taste*					
0 (Reference)					
1	**1.021**	**0.120**	**8.511**	**2.78 [2.20–3.52]**	**<0.001**
2	**1.291**	**0.128**	**10.129**	**3.64 [2.84–4.68]**	**<0.001**
3	**1.608**	**0.146**	**11.004**	**4.99 [3.76–6.66]**	**<0.001**
4 or more	**1.747**	**0.158**	**11.089**	**5.74 [4.22–7.83]**	**<0.001**
Every one more disease	**0.333**	**0.032**	**10.484**	**1.39 [1.31–1.49]**	**<0.001**

OR, odds ratio; CI, confidence interval.

Models for associations of number of diseases with COVID-19 and its symptoms were adjusted for age, gender, race, education, family income-poverty ratio, household region, health insurance hierarchy, marital status, smoking status, body mass index, history of pneumonia shot and flu vaccine in the past 12 month, and interview month. Bold values indicates that the association has statistical significance (*P*<0.05).

**Figure 2:**
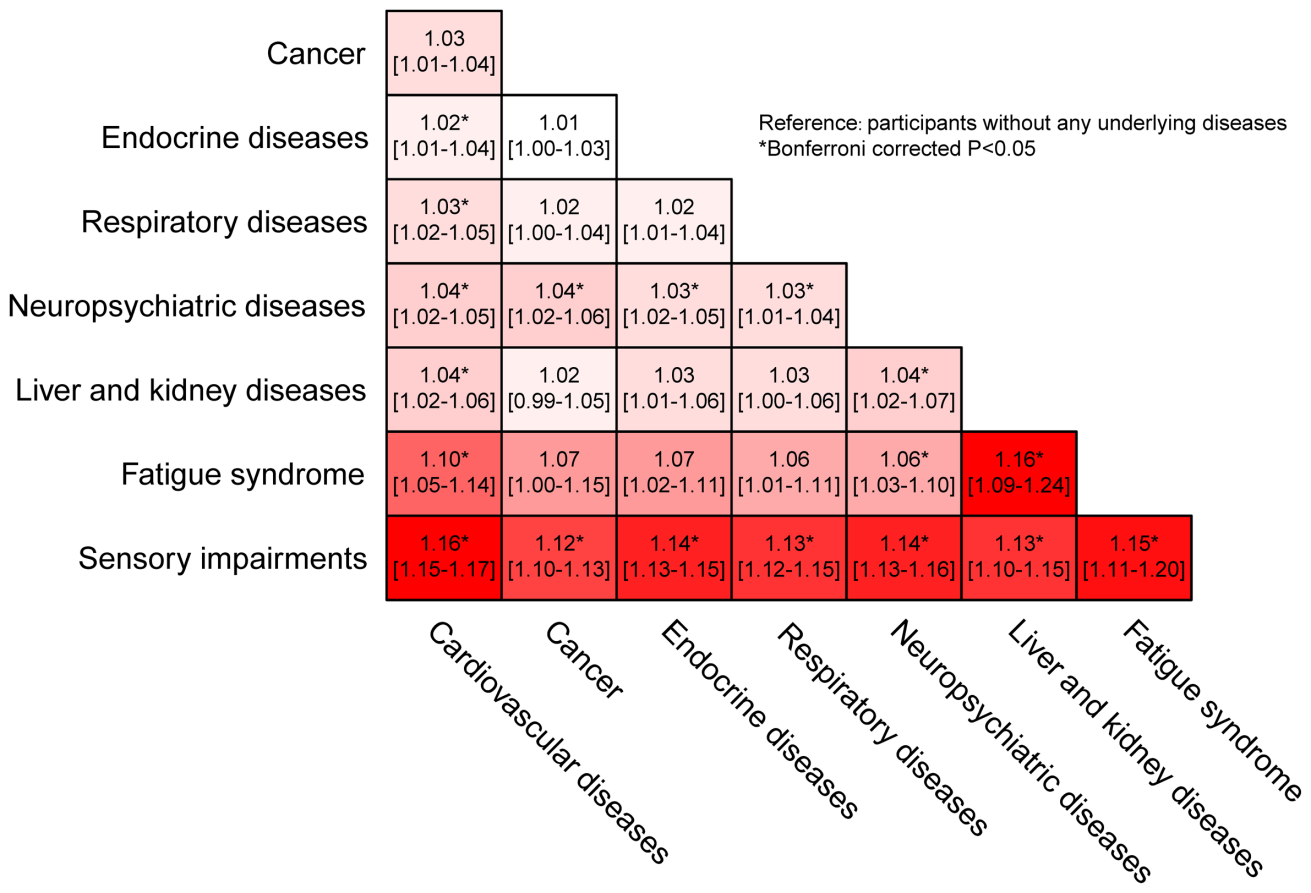
Odds ratio and 95% confidence intervals of COVID-19 according to the dyads of underlying diseases in the National Health Interview Survey. The odds ratio of dyads was adjusted for age, gender, race, education, family income-poverty ratio, household region, health insurance hierarchy, marital status, smoking status, body mass index, history of pneumonia shot and flu vaccine in the past 12 months, interview month and presence of other underlying diseases. Participants without any underlying diseases were defined as the reference group. Each block represents the relation of the combination of its corresponding disease on the horizontal and vertical axes to COVID-19. The depth reflects the strength of associations, ranging from white (the lowest odds ratio) to red (the highest odds ratio).

The associations of specific underlying diseases are presented in Table 4. In the multivariable adjusted model, besides cancer, cardiovascular diseases (OR: 1.25, 95% CI: 1.14–1.37), endocrine diseases (OR: 1.12, 95% CI: 1.03–1.23), respiratory diseases (OR: 1.14, 95% CI: 1.03–1.25), neuropsychiatric diseases (OR: 1.25, 95% CI: 1.15–1.36), liver and kidney diseases (OR: 1.28, 95% CI: 1.08–1.50), fatigue syndrome (OR: 1.60, 95% CI: 1.20–2.10), and sensory impairments (OR: 3.77, 95% CI: 3.48–4.09) were related to higher odds of COVID-19. In the mutually adjusted model, sensory impairments were independently associated with higher odds of COVID-19 (OR: 3.73, 95% CI: 3.44–4.05), severe symptoms (OR: 1.37, 95% CI: 1.13–1.67), and loss of smell (OR: 8.17, 95% CI: 6.86–9.76) and taste (OR: 6.13, 95% CI: 5.19–7.25). In addition, the independent relations of cardiovascular diseases to COVID-19 (OR: 1.13, 95% CI: 1.03–1.24), neuropsychiatric diseases to severe symptoms (OR: 1.41, 95% CI: 1.15–1.74), endocrine diseases to loss of taste were also detected (OR: 1.28, 95% CI: 1.05–1.56). The dyads most strongly associated with COVID-19 were those involving sensory impairments (Figure 2), particularly sensory impairments combined with cardiovascular diseases (OR: 1.16, 95% CI: 1.15–1.17), fatigue syndrome (OR: 1.15, 95% CI: 1.11–1.20), endocrine diseases (OR: 1.14, 95% CI: 1.13–1.15), and neuropsychiatric diseases (OR: 1.14, 95% CI: 1.13–1.16). Furthermore, dyads of fatigue syndrome with liver and kidney diseases were also relatively strongly associated with COVID-19 (OR: 1.16, 95% CI: 1.09–1.24).

**Table 4:** Associations of specific diseases with COVID-19 and its symptoms in the National Health Interview Survey.

	β	Standard error	Wald *χ*^2^	OR [95% CI]	*P*-value
*COVID-19 history*					
Multivariable adjusted model					
Cardiovascular diseases	**0.223**	**0.047**	**4.777**	**1.25 [1.14–1.37]**	**<0.001**
Cancer	0.077	0.066	1.172	1.08 [0.95–1.23]	0.241
Endocrine diseases	**0.118**	**0.046**	**2.541**	**1.12 [1.03–1.23]**	**0.011**
Respiratory diseases	**0.129**	**0.050**	**2.563**	**1.14 [1.03–1.25]**	**0.010**
Neuropsychiatric diseases	**0.224**	**0.044**	**5.066**	**1.25 [1.15–1.36]**	**<0.001**
Liver and kidney diseases	**0.245**	**0.082**	**2.965**	**1.28 [1.08–1.50]**	**0.003**
Fatigue Syndrome	**0.471**	**0.141**	**3.332**	**1.60 [1.20–2.10]**	**0.001**
Sensory impairments	**1.327**	**0.041**	**32.290**	**3.77 [3.48–4.09]**	**<0.001**
Mutually adjusted model					
Cardiovascular diseases	**0.122**	**0.049**	**2.481**	**1.13 [1.03–1.24]**	**0.013**
Cancer	0.003	0.067	0.047	1.00 [0.88–1.14]	0.962
Endocrine diseases	−0.007	0.049	−0.137	0.99 [0.90–1.09]	0.891
Respiratory diseases	0.002	0.052	0.039	1.00 [0.90–1.11]	0.969
Neuropsychiatric diseases	0.007	0.047	0.158	1.01 [0.92–1.10]	0.875
Liver and kidney diseases	0.026	0.085	0.303	1.03 [0.87–1.21]	0.762
Fatigue Syndrome	0.207	0.146	1.416	1.23 [0.92–1.63]	0.157
Sensory impairments	**1.317**	**0.042**	**31.612**	**3.73 [3.44–4.05]**	**<0.001**
*Severe symptoms*					
Multivariable adjusted model					
Cardiovascular diseases	0.149	0.107	1.392	1.16 [0.94–1.43]	0.164
Cancer	0.116	0.149	0.779	1.12 [0.84–1.50]	0.436
Endocrine diseases	**0.252**	**0.106**	**2.368**	**1.29 [1.04–1.58]**	**0.018**
Respiratory diseases	**0.240**	**0.116**	**2.071**	**1.27 [1.01–1.59]**	**0.038**
Neuropsychiatric diseases	**0.419**	**0.102**	**4.116**	**1.52 [1.24–1.85]**	**<0.001**
Liver and kidney diseases	**0.383**	**0.172**	**2.229**	**1.47 [1.04–2.05]**	**0.026**
Fatigue Syndrome	−0.077	0.310	−0.249	0.93 [0.49–1.66]	0.803
Sensory impairments	**0.362**	**0.099**	**3.649**	**1.44 [1.18–1.75]**	**<0.001**
Mutually adjusted model					
Cardiovascular diseases	0.041	0.112	0.363	1.04 [0.84–1.30]	0.717
Cancer	0.073	0.151	0.483	1.08 [0.80–1.44]	0.629
Endocrine diseases	0.171	0.111	1.545	1.19 [0.95–1.47]	0.122
Respiratory diseases	0.164	0.118	1.385	1.18 [0.93–1.48]	0.166
Neuropsychiatric diseases	**0.346**	**0.105**	**3.289**	**1.41 [1.15–1.74]**	**0.001**
Liver and kidney diseases	0.275	0.178	1.544	1.32 [0.92–1.86]	0.123
Fatigue Syndrome	−0.403	0.321	−1.257	0.67 [0.35–1.22]	0.209
Sensory impairments	**0.317**	**0.100**	**3.164**	**1.37 [1.13–1.67]**	**0.002**
*Loss of smell*					
Multivariable adjusted model					
Cardiovascular diseases	0.091	0.089	1.020	1.10 [0.92–1.31]	0.308
Cancer	−0.212	0.129	−1.644	0.81 [0.63–1.04]	0.100
Endocrine diseases	0.143	0.089	1.602	1.15 [0.97–1.38]	0.109
Respiratory diseases	−0.121	0.098	−1.241	0.89 [0.73–1.07]	0.215
Neuropsychiatric diseases	0.010	0.085	0.114	1.01 [0.85–1.19]	0.909
Liver and kidney diseases	0.161	0.160	1.006	1.18 [0.86–1.61]	0.314
Fatigue Syndrome	0.156	0.273	0.572	1.17 [0.69–2.02]	0.568
Sensory impairments	**2.064**	**0.089**	**23.277**	**7.88 [6.63–9.39]**	**<0.001**
Mutually adjusted model					
Cardiovascular diseases	0.041	0.104	0.394	1.04 [0.85–1.28]	0.694
Cancer	−0.237	0.144	−1.648	0.79 [0.59–1.05]	0.099
Endocrine diseases	0.124	0.103	1.199	1.13 [0.92–1.39]	0.230
Respiratory diseases	**−0.247**	**0.110**	**−2.234**	**0.78 [0.63–0.97]**	**0.025**
Neuropsychiatric diseases	−0.226	0.098	−2.298	0.80 [0.66–0.97]	0.022
Liver and kidney diseases	−0.039	0.182	−0.211	0.96 [0.67–1.38]	0.833
Fatigue Syndrome	0.070	0.305	0.228	1.07 [0.59–1.97]	0.820
Sensory impairments	**2.100**	**0.090**	**23.313**	**8.17 [6.86–9.76]**	**<0.001**
*Loss of taste*					
Multivariable adjusted model					
Cardiovascular diseases	0.135	0.089	1.516	1.14 [0.96–1.36]	0.130
Cancer	−0.117	0.128	−0.915	0.89 [0.69–1.14]	0.360
Endocrine diseases	**0.267**	**0.089**	**3.001**	**1.31 [1.10–1.56]**	**0.003**
Respiratory diseases	−0.089	0.096	−0.929	0.91 [0.76–1.10]	0.353
Neuropsychiatric diseases	0.125	0.084	1.484	1.13 [0.96–1.34]	0.138
Liver and kidney diseases	0.313	0.160	1.955	1.37 [1.00–1.88]	0.051
Fatigue Syndrome	0.285	0.273	1.045	1.33 [0.79–2.30]	0.296
Sensory impairments	**1.803**	**0.084**	**21.412**	**6.07 [5.15–7.16]**	**<0.001**
*Mutually adjusted model*					
Cardiovascular diseases	0.043	0.100	0.428	1.04 [0.86–1.27]	0.669
Cancer	−0.147	0.139	−1.057	0.86 [0.66–1.13]	0.291
Endocrine diseases	**0.245**	**0.100**	**2.449**	**1.28 [1.05–1.56]**	**0.014**
Respiratory diseases	**−0.215**	**0.107**	**−2.019**	**0.81 [0.65–0.99]**	**0.043**
Neuropsychiatric diseases	−0.078	0.095	−0.822	0.93 [0.77–1.11]	0.411
Liver and kidney diseases	0.099	0.178	0.557	1.10 [0.78–1.57]	0.578
Fatigue Syndrome	0.118	0.302	0.391	1.13 [0.63–2.06]	0.695
Sensory impairments	**1.813**	**0.085**	**21.310**	**6.13 [5.19–7.25]**	**<0.001**

OR, odds ratio; CI, confidence interval.

Multivariable adjusted models for associations of each disease with COVID-19 and its symptoms were adjusted for age, gender, race, education, family income-poverty ratio, household region, health insurance hierarchy, marital status, smoking status, body mass index, history of pneumonia shot and flu vaccine in the past 12 month, and interview month.

Mutually adjusted models were adjusted for all diseases simultaneously to allow for mutual adjustments. Bold values indicates that the association has statistical significance (*P*<0.05).

### Subgroup analyses

Subgroup analyses were conducted by age, gender, race, education, family income-poverty ratio, and marital status (Table 5), and the results were generally consistent. Moreover, the associations of number of underlying diseases with COVID-19 were strengthened in participants who were married or partnered than those who were not (P-interaction = 0.041). Among younger participants, the number of underlying diseases was more profoundly related to loss of smell (P-interaction = 0.021). No other covariates significantly interacted with number of underlying diseases in relation to COVID-19 outcomes.

**Table 5:** Subgroup associations of the number of underlying diseases with COVID-19 and its symptoms stratified by selected characteristics in the NHIS.

	Number of sensory impairments					Ever one more UD	*P*-trend	*P*-interaction
	Without UD	With one UD	With two UDs	With three UDs	With four or more UDs			
*COVID-19 history*								
Age, years								0.203
<65	1.00 [Reference]	2.21 [1.96–2.50]	2.90 [2.54–3.31]	3.50 [2.99–4.10]	3.88 [3.24–4.65]	1.36 [1.31–1.41]	<0.001	
> = 65	1.00 [Reference]	1.72 [1.09–2.80]	1.83 [1.19–2.94]	2.33 [1.52–3.74]	3.09 [2.02–4.95]	1.22 [1.14–1.29]	<0.001	
Gender								0.589
Male	1.00 [Reference]	2.20 [1.86–2.61]	2.53 [2.10–3.05]	3.07 [2.48–3.81]	3.89 [3.08–4.93]	1.32 [1.26–1.38]	<0.001	
Female	1.00 [Reference]	2.23 [1.90–2.63]	3.06 [2.59–3.64]	3.69 [3.04–4.48]	4.23 [3.45–5.20]	1.34 [1.28–1.39]	<0.001	
Race, %								0.222
Hispanic	1.00 [Reference]	2.40 [1.90–3.04]	2.69 [2.06–3.51]	3.46 [2.47–4.82]	4.38 [3.06–6.27]	1.37 [1.28–1.47]	<0.001	
Non-Hispanic White	1.00 [Reference]	2.03 [1.74–2.37]	2.66 [2.27–3.13]	3.12 [2.61–3.74]	3.78 [3.12–4.60]	1.31 [1.26–1.35]	<0.001	
Non-Hispanic Black	1.00 [Reference]	2.53 [1.75–3.70]	3.31 [2.22–4.99]	3.91 [2.46–6.22]	4.25 [2.60–6.95]	1.33 [1.22–1.46]	<0.001	
Other races	1.00 [Reference]	2.61 [1.68–4.12]	3.85 [2.39–6.27]	5.98 [3.41–10.48]	7.15 [3.78–13.42]	1.51 [1.34–1.71]	<0.001	
Education								0.582
Below college	1.00 [Reference]	2.26 [1.84–2.77]	2.84 [2.29–3.53]	3.15 [2.47–4.02]	3.75 [2.90–4.85]	1.27 [1.21–1.34]	<0.001	
College and above	1.00 [Reference]	2.19 [1.89–2.53]	2.81 [2.41–3.28]	3.52 [2.95–4.21]	4.30 [3.54–5.22]	1.36 [1.31–1.41]	<0.001	
Family income-poverty ratio, %								0.163
<1.5	1.00 [Reference]	1.66 [1.27–2.19]	2.68 [2.02–3.57]	3.30 [2.39–4.55]	3.82 [2.77–5.28]	1.32 [1.24–1.41]	<0.001	
1.5–3.5	1.00 [Reference]	2.61 [2.09–3.26]	3.25 [2.59–4.11]	4.20 [3.24–5.45]	4.62 [3.50–6.12]	1.34 [1.27–1.41]	<0.001	
>3.5	1.00 [Reference]	2.26 [1.92–2.67]	2.75 [2.30–3.28]	3.09 [2.52–3.80]	4.14 [3.29–5.22]	1.34 [1.28–1.40]	<0.001	
Married/Partnered								0.041
No	1.00 [Reference]	1.95 [1.64–2.33]	2.38 [1.97–2.88]	2.88 [2.32–3.57]	3.35 [2.67–4.20]	1.27 [1.21–1.33]	<0.001	
Yes	1.00 [Reference]	2.44 [2.08–2.87]	3.20 [2.71–3.80]	3.85 [3.17–4.66]	4.76 [3.85–5.88]	1.38 [1.32–1.44]	<0.001	
*Severe symptoms*								
Age, years								0.680
<65	1.00 [Reference]	1.26 [0.88–1.83]	2.01 [1.40–2.92]	2.13 [1.43–3.21]	2.77 [1.80–4.30]	1.23 [1.14–1.33]	<0.001	
> = 65	1.00 [Reference]	1.21 [0.36–4.94]	1.07 [0.33–4.27]	1.48 [0.48–5.83]	1.59 [0.51–6.24]	1.10 [0.95–1.28]	0.109	
Gender								0.578
Male	1.00 [Reference]	1.41 [0.85–2.40]	2.18 [1.31–3.73]	1.81 [1.02–3.26]	2.60 [1.45–4.76]	1.19 [1.07–1.33]	0.002	
Female	1.00 [Reference]	1.21 [0.75–1.98]	1.64 [1.03–2.66]	2.20 [1.35–3.65]	2.54 [1.53–4.30]	1.21 [1.10–1.32]	<0.001	
Race, %								0.489
Hispanic	1.00 [Reference]	1.14 [0.62–2.16]	1.48 [0.76–2.92]	2.82 [1.38–5.85]	2.37 [1.07–5.30]	1.22 [1.04–1.42]	0.015	
Non-Hispanic White	1.00 [Reference]	1.48 [0.89–2.54]	2.26 [1.39–3.83]	2.37 [1.42–4.11]	2.94 [1.72–5.18]	1.21 [1.11–1.33]	<0.001	
Non-Hispanic Black	1.00 [Reference]	1.42 [0.53–4.15]	1.46 [0.52–4.40]	0.65 [0.19–2.29]	2.76 [0.83–9.80]	1.16 [0.92–1.45]	0.214	
Other races	1.00 [Reference]	0.68 [0.15–3.31]	2.45 [0.62–11.25]	2.51 [0.48–14.42]	3.80 [0.77–21.28]	1.40 [1.00–1.98]	0.053	
Education								0.287
Below college	1.00 [Reference]	1.61 [0.95–2.81]	1.73 [1.01–3.03]	2.04 [1.15–3.72]	3.08 [1.69–5.72]	1.23 [1.10–1.37]	<0.001	
College and above	1.00 [Reference]	1.09 [0.69–1.76]	1.97 [1.27–3.14]	2.04 [1.26–3.36]	2.32 [1.41–3.89]	1.18 [1.08–1.30]	<0.001	
Family income-poverty ratio, %								0.781
<1.5	1.00 [Reference]	2.13 [1.03–4.66]	2.79 [1.36–6.06]	3.31 [1.55–7.46]	4.36 [2.01–9.98]	1.23 [1.08–1.41]	0.002	
1.5–3.5	1.00 [Reference]	1.05 [0.58–1.93]	1.54 [0.86–2.83]	1.59 [0.86–3.04]	1.66 [0.86–3.27]	1.13 [1.01–1.28]	0.004	
>3.5	1.00 [Reference]	1.13 [0.67–1.98]	1.69 [1.00–2.96]	1.86 [1.04–3.40]	2.85 [1.55–5.35]	1.27 [1.13–1.43]	<0.001	
Married/Partnered								0.469
No	1.00 [Reference]	1.48 [0.89–2.51]	1.76 [1.05–3.01]	2.24 [1.30–3.94]	2.26 [1.29–4.05]	1.14 [1.03–1.27]	0.001	
Yes	1.00 [Reference]	1.18 [0.73–1.93]	1.95 [1.24–3.17]	1.88 [1.14–3.16]	2.96 [1.76–5.08]	1.26 [1.14–1.39]	<0.001	
*Loss of smell*								
Age, years								0.021
<65	1.00 [Reference]	2.74 [2.16–3.50]	3.95 [3.04–5.16]	4.32 [3.16–5.93]	6.17 [4.28–8.95]	1.45 [1.35–1.56]	<0.001	
> = 65	1.00 [Reference]	3.95 [1.36–13.53]	2.14 [0.75–7.22]	5.05 [1.79–16.98]	4.23 [1.50–14.21]	1.19 [1.05–1.36]	0.008	
Gender								0.649
Male	1.00 [Reference]	3.13 [2.21–4.45]	4.01 [2.75–5.89]	4.44 [2.89–6.90]	5.13 [3.21–8.27]	1.34 [1.22–1.48]	<0.001	
Female	1.00 [Reference]	2.74 [1.98–3.80]	3.47 [2.47–4.90]	4.90 [3.33–7.28]	5.53 [3.65–8.43]	1.40 [1.28–1.52]	<0.001	
Race, %								0.808
Hispanic	1.00 [Reference]	2.33 [1.47–3.70]	4.02 [2.32–7.09]	4.32 [2.22–8.69]	4.51 [2.22–9.43]	1.43 [1.23–1.67]	<0.001	
Non-Hispanic White	1.00 [Reference]	3.60 [2.61–4.99]	4.00 [2.88–5.61]	5.44 [3.75–7.94]	6.52 [4.37–9.81]	1.38 [1.27–1.49]	<0.001	
Non-Hispanic Black	1.00 [Reference]	2.69 [1.22–6.08]	4.10 [1.74–10.00]	4.24 [1.65–11.36]	10.89 [3.64–34.50]	1.58 [1.28–1.97]	<0.001	
Other races	1.00 [Reference]	1.99 [0.71–5.87]	3.92 [1.36–12.03]	4.58 [1.25–18.13]	1.71 [0.45–6.64]	1.17 [0.88–1.55]	0.277	
Education								0.521
Below college	1.00 [Reference]	2.82 [1.88–4.26]	2.92 [1.91–4.49]	4.97 [3.05–8.20]	4.82 [2.88–8.16]	1.32 [1.19–1.47]	<0.001	
College and above	1.00 [Reference]	2.94 [2.19–3.96]	4.07 [2.98–5.60]	4.62 [3.23–6.65]	5.78 [3.91–8.60]	1.41 [1.30–1.53]	<0.001	
Family income-poverty ratio, %								0.098
<1.5	1.00 [Reference]	2.07 [1.18–3.66]	3.13 [1.73–5.75]	2.73 [1.43–5.29]	6.02 [3.00–12.34]	1.36 [1.18–1.56]	<0.001	
1.5–3.5	1.00 [Reference]	2.41 [1.56–3.77]	3.18 [2.01–5.10]	4.82 [2.87–8.21]	5.65 [3.23–10.01]	1.40 [1.25–1.57]	<0.001	
>3.5	1.00 [Reference]	3.72 [2.66–5.25]	4.44 [3.09–6.44]	6.05 [3.96–9.35]	5.70 [3.58–9.15]	1.41 [1.29–1.56]	<0.001	
Married/Partnered								0.907
No	1.00 [Reference]	2.94 [2.08–4.20]	3.44 [2.36–5.03]	4.30 [2.80–6.67]	5.47 [3.46–8.70]	1.36 [1.24–1.50]	<0.001	
Yes	1.00 [Reference]	2.80 [2.03–3.88]	3.81 [2.71–5.37]	5.17 [3.51–7.68]	5.37 [3.52–8.26]	1.40 [1.28–1.52]	<0.001	
*Loss of taste*								
Age, years								0.492
<65	1.00 [Reference]	2.68 [2.11–3.41]	3.73 [2.87–4.85]	4.78 [3.50–6.56]	5.91 [4.13–8.53]	1.44 [1.34–1.55]	<0.001	
> = 65	1.00 [Reference]	5.49 [1.61–26.47]	4.14 [1.24–19.77]	8.25 [2.49–39.30]	8.22 [2.48–39.17]	1.28 [1.13–1.47]	<0.001	
Gender								0.870
Male	1.00 [Reference]	3.30 [2.33–4.71]	4.22 [2.89–6.20]	5.80 [3.76–9.04]	6.60 [4.12–10.68]	1.43 [1.30–1.58]	<0.001	
Female	1.00 [Reference]	2.49 [1.80–3.45]	3.27 [2.33–4.60]	4.50 [3.06–6.65]	5.06 [3.35–7.68]	1.36 [1.25–1.48]	<0.001	
Race, %								0.180
Hispanic	1.00 [Reference]	2.24 [1.43–3.54]	4.15 [2.43–7.21]	3.36 [1.79–6.43]	4.16 [2.09–8.46]	1.38 [1.20–1.61]	<0.001	
Non-Hispanic White	1.00 [Reference]	3.41 [2.47–4.73]	4.65 [3.33–6.53]	6.16 [4.25–9.02]	8.06 [5.39–12.17]	1.45 [1.34–1.57]	<0.001	
Non-Hispanic Black	1.00 [Reference]	2.73 [1.25–6.10]	1.54 [0.67–3.58]	4.65 [1.78–12.68]	3.75 [1.31–11.05]	1.28 [1.05–1.57]	0.018	
Other races	1.00 [Reference]	2.10 [0.69–6.68]	4.21 [1.38–13.77]	10.99 [2.76–48.92]	5.22 [1.29–23.34]	1.50 [1.11–2.05]	0.001	
Education								0.767
Below college	1.00 [Reference]	3.36 [2.23–5.10]	3.90 [2.54–6.03]	6.17 [3.78–10.19]	5.88 [3.50–9.98]	1.37 [1.23–1.52]	<0.001	
College and above	1.00 [Reference]	2.60 [1.95–3.49]	3.69 [2.71–5.05]	4.74 [3.32–6.80]	5.93 [4.02–8.81]	1.42 [1.32–1.54]	<0.001	
Family income-poverty ratio, %								0.132
<1.5	1.00 [Reference]	2.19 [1.26–3.80]	3.10 [1.74–5.58]	3.28 [1.74–6.29]	5.17 [2.64–10.33]	1.35 [1.18–1.54]	<0.001	
1.5–3.5	1.00 [Reference]	2.25 [1.46–3.49]	2.93 [1.86–4.65]	5.39 [3.22–9.14]	5.23 [3.02–9.17]	1.38 [1.24–1.54]	<0.001	
>3.5	1.00 [Reference]	3.50 [2.50–4.94]	4.76 [3.31–6.92]	6.12 [4.01–9.46]	7.40 [4.63–11.96]	1.50 [1.36–1.65]	<0.001	
Married/Partnered								0.983
No	1.00 [Reference]	2.66 [1.88–3.80]	3.31 [2.27–4.86]	4.59 [2.98–7.13]	5.22 [3.30–8.31]	1.36 [1.24–1.50]	<0.001	
Yes	1.00 [Reference]	2.98 [2.16–4.12]	4.07 [2.91–5.73]	5.61 [3.83–8.30]	6.70 [4.39–10.30]	1.44 [1.33–1.57]	<0.001	

NHIS: National Health Interview Survey, UD: underlying disease, CI: confidence interval, OR: odds ratio.

All models were adjusted for age, gender, race, education, family income-poverty ratio, household region, health insurance hierarchy, marital status, smoking status, body mass index, history of pneumonia shot and flu vaccine in the past 12 month, and interview month.

## Discussion

The nationwide study detected dose–response associations of larger numbers of underlying diseases with higher odds of COVID-19, severe symptoms, loss of smell, and loss of taste. Each additional disease was related to 33% higher odds of COVID-19. Specific diseases were independently associated with COVID-19 and its symptoms. The associations were generally consistent across the study subgroups. The findings support that individuals with poorer health conditions are more likely to become infected with COVID-19 and have more severe post-infection symptoms.

Previous studies also reported that COVID-19 patients were more likely to have underlying diseases. For instance, Onder et al. reported that most COVID-19 patients in Italy had underlying diseases, 25.6% had 2 underlying diseases, 48.5% had 3 or more underlying diseases, and multiple comorbidities account for a larger proportion of infection and severe symptoms (21), which was consistent with the present study. The findings echoed the established knowledge that pre-existing worse health conditions might be related to severe COVID-19 outcomes (13, 22, 23). According to a meta-analysis of 160 studies in North America, Europe, and the Western Pacific (13), pre-existing diabetes (hazard ratio [HR] ranged 1.2–2.0), heart failure (HR ranged 1.3–3.3), COPD (HR ranged 1.12–2.2), dementia (HR ranged 1.4–7.7), liver cirrhosis (OR ranged 3.2–5.9) were related to higher risk of severe COVID-19 symptoms and deaths, which was generally confirmed by findings in the present study. A relation of active cancer to worse COVID-19 outcomes was also detected in the study, which was not found in this study, possibly because the study restricted general cancer patients, including active cancers and other types. Further studies on different types of cancer patients are needed. The study confirmed previous research in related fields and provided more evidence on the relationship of underlying diseases such as neuropsychological disorders, fatigue syndrome, and sensory impairments to COVID-19 and its symptoms. Furthermore, present findings suggest that dyads involving sensory impairments were particularly associated with higher odds of COVID-19, and married or partnered participants with underlying diseases may be more likely to be infected. There findings may provide evidence for prevention work of key populations.

Although the typical mechanisms are not well understood, the observed associations could be explained by several possible pathways, including dysregulation of immune responses, pro-inflammatory environments, high expression of ACE2 receptors, and neuroinflammation. SARS-CoV-2 enters the cell by attaching to the ACE2 receptor on the cell surface with its spike protein S (1). ACE2 receptors are highly expressed in adipose tissue (24) and are more active in diabetics (25), which may promote viral invasion. The pro-inflammatory environment in patients with underlying diseases like pre-existing cardiovascular and endocrine disorders will be more likely to have adverse outcomes such as cytokine storms, immune system disorders, endothelial cell destruction, thrombosis-related inflammation, and an imbalance in oxygen supply and demand, causing damage to multiple organs and severe symptoms (26–28). The decline in ACE2 activity during viral infection might exacerbate the renin–angiotensin–aldosterone system (RAAS) dysfunction, altering the body’s fluid/electrolyte balance and blood pressure, aggravating infection symptoms (29, 30). Several studies have shown that cancer patients have a higher susceptibility to COVID-19 and adverse outcomes (31, 32), which are related to the suppression of the immune response, radiotherapy, and chemotherapy (33). When people have multiple underlying conditions, which is particularly common in the older adult, their bodies may be affected by many of these factors at the same time, leading to a higher likelihood of infection and more severe symptoms. This research also found that neuropsychological illness is a risk factor for severe COVID-19. Research on neuropsychiatric disorders has discovered that Alzheimer’s disease (AD) and all-cause dementia are age-independent risk factors for disease severity and mortality in COVID-19 (34, 35). Since neuronal endothelium express ACE2 receptors, the virus can infect the human nervous system directly (36). As a result, in the context of neuropsychiatric and sensory disorders, acute and secondary immunological alterations could affect these individuals more severely (37). According to previous studies (38–40), the prevalence of COVID-19 can lead to interruptions in rehabilitation training and recovery, and also decrease physical activity. Patients would have an increase in neuropsychiatric disorder symptoms as a result of this transition, which may have an impact on their physical functioning and interact with psychosocial factors to contribute to COVID-19 symptoms. Meanwhile, Care facilities may provide long-term care to patients with underlying diseases. These human-intensive living environments are more prone to infectious disease outbreaks, and the underlying clinical characteristics of these patients may increase susceptibility and disease severity rates.

In the context of COVID-19 pandemic, the present study has both public health and social significance. As mentioned earlier, people with underlying diseases are more likely to develop severe symptoms after infection, leading to higher hospital admissions and medical treatment needs (7, 41). Approximately 22% of people worldwide currently have at least one underlying medical condition and are at high odds of developing severe COVID-19 (7). A significant portion of the additional healthcare burden of the COVID-19 pandemic is caused by infections in people with underlying medical conditions (41). In responding to the COVID-19 pandemic, structural inequalities in public health resources may contribute to the development of such underlying conditions or exacerbations in pre-existing populations (42–44). Comprehensive health conditions and underlying diseases need to be taken into consideration when formulating prevention strategies (45). Given the limited medical resources (46), people with multiple underlying diseases need to be relatively prioritized for inclusion in vaccine protection to reduce the odds of infection, alleviate symptoms after infection, and lessen the social healthcare burden. It could be necessary to scale up certain health measures, such as improving surveillance and basic healthcare and incorporating people and families in the management of chronic diseases (47–49).

The strengths of the present study were the large and national sample and the relatively comprehensive consideration of underlying diseases. However, it should be cautiously interpreted the findings due to some limitations. Because of the cross-sectional nature of this study, reverse causality might exist. Given the long latency of underlying diseases the study focused on Allegrante et al. (50), participants may already have functional decline before diagnosis. It may help to explain the impact of functional states of various organ systems on COVID-19 and its symptoms as the short duration of the COVID-19 pandemic, although the chronological order of underlying diseases and COVID-19 is difficult to determine. Secondly, since self-reporting of some diseases might underestimate the actual prevalence (51), measurement error may be inevitable. The lack of studies on reliability of self-reported underlying diseases in NHIS was another limitation, although some of them have been validated in other studies (52–55). Further research on more comprehensive and clearly diagnosed diseases is needed. Additionally, it was difficult to define symptoms precisely. For instance, opinions on the severity of COVID-19 varied between individuals and participants in poor health may accurately report their conditions. Fourth, residual confounding may not be fully considered. Owing to lack of related information, physical activity and drinking status were not able to be adjusted for in the NHIS. In addition, due to the lack of information on SARS-CoV-2 variants, it was unable to assess the consistency in subgroup infected with different variants. Relevant population studies are needed since there are variances in COVID-19 outcomes brought on by various variants (56). Finally, because the study was based on the US population, whether it applies to other populations warrants further confirmation.

## Conclusion

In this nationwide study, dose–response relations of a larger number of underlying diseases to higher odds of COVID-19, severe symptoms, loss of smell, and loss of taste were detected. Specific underlying diseases might be individually associated with COVID-19 and its symptoms. Although COVID-19 is largely under control worldwide, people with multiple underlying diseases should receive adequate attention, and precautions should be taken to lessen the risk of infection and potential serious consequences.

## Data availability statement

Publicly available datasets were analyzed in this study. This data can be found at: NHIS 2021 (https://www.cdc.gov/nchs/nhis/data-questionnaires-documentation.htm).

## Ethics statement

The NHIS was approved by the research ethics review board of the National Center for Health Statistics of the US, Centers for Disease Control and Prevention (CDC). Oral or written informed consent was provided by all participants. The patients/participants provided their written informed consent to participate in this study.

## Author contributions

XL and BW designed the study. BW performed the statistical analyses. BW and SY interpreted the data and drafted the manuscript. HL, SR, YL, and XN further revised the manuscript. XL supervised the data analysis and interpretation and the primary responsibility for the study final content. All authors contributed to the article and approved the submitted version.

## Conflict of interest

The authors declare that the research was conducted in the absence of any commercial or financial relationships that could be construed as a potential conflict of interest.

## Publisher’s note

All claims expressed in this article are solely those of the authors and do not necessarily represent those of their affiliated organizations, or those of the publisher, the editors and the reviewers. Any product that may be evaluated in this article, or claim that may be made by its manufacturer, is not guaranteed or endorsed by the publisher.

## Footnotes

^1^ https://www.cdc.gov/nchs/nhis/index.htm
